# FUT1-mediated terminal fucosylation acts as a new target to attenuate renal fibrosis

**DOI:** 10.1186/s10020-023-00639-0

**Published:** 2023-04-21

**Authors:** Jialiang Luo, Kaifeng Mao, Zhengyumeng Zhu, Junli Ye, Lei Li, Di Wang, Jia Zhou, Fenwang Lin, Juan Li, Junsheng Ye

**Affiliations:** 1 Department of Dermatology, the Fifth Affiliated Hospital, Southern Medical University, Guangzhou, Guangdong 510900 China; 2 Department of Kidney Transplantation, Nanfang Hospital, Southern Medical University, Guangzhou, Guangdong China; 3 Department of Medical Laboratory, School of Laboratory Medicine and Biotechnology, Southern Medical University, Guangzhou, Guangdong China; 4 Department of Physiology and Pathophysiology, School of Basic Medicine, Medical College, Qingdao University, Qingdao, China; 5 Department of Dermatology, Dermatology Hospital of Southern Medical University, Southern Medical University, Guangzhou, Guangdong China; 6 Department of Immunology, School of Basic Medical Sciences, Southern Medical University, Guangzhou, Guangdong China; 7 Department of Kidney Transplantation, Beijing Tsinghua Changgung Hospital, School of Clinical Medicine, Tsinghua University, Beijing, 102218 China; 8 School of Nursing, Southern Medical University, Guangzhou, Guangdong 510900 China

**Keywords:** Terminal fucosylation, Renal fibrosis, 2-D-gal, EMT, TGF-β/Smad signaling

## Abstract

**Backgrounds:**

Renal fibrosis is a common pathologic process of most chronic kidney diseases (CKDs), becoming one of the major public health problems worldwide. Terminal fucosylation plays an important role in physiological homeostasis and pathological development. The present study aimed to explore the role of terminal fucosylation during kidney fibrogenesis and propose a possible anti-fibrosis treatment via suppressing aberrant terminal fucosylation.

**Methods:**

We investigated the expression level of fucosyltransferase1 (FUT1) in CKD patients by using public database. Then, we further confirmed the level of terminal fucosylation by UEA-I staining and FUT1 expression in unilateral ureteral obstruction (UUO)-induced renal fibrosis mice. Immunostaining, qPCR, western blotting and wound healing assay were applied to reveal the effect of FUT1 overexpression in human kidney proximal tubular epithelial cell (HK-2). What’s more, we applied terminal fucosylation inhibitor, 2-Deoxy-D-galactose (2-D-gal), to determine whether suppressing terminal fucosylation ameliorates renal fibrosis progression in vitro and in vivo.

**Results:**

Here, we found that the expression of FUT1 significantly increased during renal fibrosis. In vitro experiments showed upregulation of epithelial-mesenchymal transition (EMT) after over-expression of FUT1 in HK-2. Furthermore, in vivo and in vitro experiments indicated that suppression of terminal fucosylation, especially on TGF-βR I and II, could alleviate fibrogenesis via inhibiting transforming growth factor-β (TGF-β)/Smad signaling.

**Conclusions:**

The development of kidney fibrosis is attributed to FUT1-mediated terminal fucosylation, shedding light on the inhibition of terminal fucosylation as a potential therapeutic treatment against renal fibrosis.

**Supplementary Information:**

The online version contains supplementary material available at 10.1186/s10020-023-00639-0.

## Introduction

Renal fibrosis is a universal pathogenesis engaged with numerous chronic kidney diseases (CKDs) [1, 2]. In recent years, CKD is becoming as a global public health problem because of the high prevalence among general population and limited approaches to attenuate or ameliorate renal fibrogenesis [3, 4]. The progression of renal fibrosis is a multifactorial process characterized by persistent inflammation and excessive deposition of extracellular matrix (ECM), leading to irreversible destruction of normal kidney structure in the end. Notedly, accumulation of interstitial ECM under pathological conditions is attributed to activated fibroblasts [5] and 30% of fibroblasts are derived via epithelial mesenchymal transition (EMT) from the tubular epithelial cells [6, 7]. Among of multiple stimulus for EMT induction, transforming growth factor-β1 (TGF-β1) is generally considered as the chief pathogenic driver, majorly activating TGFβ/Smad signaling to promote EMT in transcriptional level via the interaction with TGF-βR I and TGF-βR II [8]. Therefore, targeting TGFβ/Smad signaling has been considered a promising therapeutic treatment to attenuate or reverse renal fibrogenesis over the past decade.

Fucosylation, a significant post-translational modification, regulates protein function and gets involved with multiple cellular processes, including signaling, recognition and cell matrix interaction [9, 10]. Mediated by the coordination of various fucosyltransferases (FUTs), fucosylation has been reported to be closely associated with the development of organ fibrosis. Shen et al. have found that FUT8 upregulated core fucosylation on TGF-β receptor in unilateral ureteral obstruction (UUO) rats and inhibition of core fucosylation by shRNA attenuated the progression of renal fibrosis in vivo [11]. What’s more, diabetic kidney injury was ameliorated by FUT8 mutation, accompanied with the downregulation of Smad and ERK signaling pathways [12]. Nevertheless, the role of terminal fucosylation mediated mainly by FUT1 in kidney fibrosis and CKD remains unclear to date.

It is well-known that 2-Deoxy-D-galactose (2-D-gal) is a terminal fucosylation inhibitor, remarkedly impeding glycoprotein synthesis. Interestingly, accumulating evidence indicated inhibition of terminal fucosylation by the application of 2-D-gal is beneficial to multiple diseases, including alleviating rheumatoid arthritis [13], dry eye disease [14] and prolonging skin transplantation [15], driving us to uncover the possible utilization of 2-D-gal against renal fibrosis.

In this study, we first observed FUT1-mediated terminal fucosylation was upregulated in CKD and renal fibrosis. Furthermore, inhibition of terminal fucosylation by 2-D-gal exerts anti-fibrotic via TGF-β/Smad signaling pathway in TGF-β1-stimulated HK-2 cell and unilateral ureteral obstruction (UUO) mouse model. In summary, our current findings indicate that terminal fucosylation promotes the progression of renal fibrosis, and the inhibition of terminal fucosylation, such as 2-D-gal, is an effective intervention against renal fibrosis via inhibiting TGF-βR fucosylation and ultimately EMT process.

## Materials and methods

### Reagents and antibodies

FITC-conjugated goat anti-rabbit IgG (ab6717), FITC-conjugated goat anti-mouse IgG (ab6785), Cy3®-conjugated goat anti-mouse IgG (ab97035), Cy3®-conjugated goat anti-rabbit IgG (ab6939), horseradish peroxidase (HRP)-conjugated goat anti-rabbit IgG (ab6721), horseradish peroxidase (HRP)-conjugated goat anti-mouse IgG (ab6789), anti-Fibronectin (ab2413) were all from Abcam (UK). For western blot, antibody against Smad2/3 (8685), p-Smad2 (18338), p-Smad3 (9520), α-SMA (19245), collagen I (81375), E-cadherin (3195), N-cadherin (13116), Vimentin (46173), Slug (9585) and Snail (3879) were obtained from Cell Signaling Technology (USA). Anti-FUT1 (17956-1-AP), anti-TGF-βR II (66636-1-Ig) and anti-GAPDH (60004-1-Ig) were supplied by Proteintech (USA). Antibody against TGF-βR I (A0708) was purchased from ABclonal (China). Ulex europaeus agglutinin-I (UEA-I) UEA-I (B-1065-2) was purchased from Vector laboratories (USA). UEA-I (L32476) was bought from Thermal Fisher (USA). TGF-β1 (100 − 21) was from PeproTech (USA). 2-D-gal (259580) was purchased from Merck (USA). LY2109761 (S2704) was obtained from Selleck Chemicals (USA).

### Animals

Wild-type C57BL/6 (6–8 weeks) male mice were purchased from the Animal Center of Southern Medical University. Animals were allowed free access to standard chow and water, housed in a specific pathogen-free animal condition with a controlled temperature (20–25 °C) and humidity (50 ± 5%). All animal experiments were approved by the Welfare and Ethical Committee for Experimental Animal Care of Southern Medical University.

The UUO mouse model was established based on the methods of previous study [16, 17]. In brief, the abdominal cavity was exposed by a midline incision and the left ureter was isolated and ligated. The sham group only received incision as a control without ureter ligation. After surgery, mice were intraperitoneally injected with 2-D-gal (250 mg/kg body weight) or equivalent saline every other day until day 14.

### Cell culture

The human HK-2 cells were grown and maintained in Dulbecco’s modified Eagle’s medium (DMEM) supplemented with 10% heat-inactivated fetal bovine serum (FBS) in a humidified chamber with 5% CO_2_ at 37 °C.

HK-2 cells were treated with 2-D-gal (0.6, 1.2 or 2.4 mM) and TGF-β1 (10 ng/mL) for 48 h. In some experiments, HK-2 was treated with LY2109761 (10 µM) 2 h ahead of indicated stimulation.

### Plasmid overexpression

Plasmid was synthesized by Beijing Genomics institution (China) and was used to overexpress FUT1 expression in HK-2 cells. Control vector was used to as a control to exclude possible non-specific effects of interference. Before other indicated experiments, cells were transfected with plasmid by lipo3000 reagent following to the standard protocol provided by manufacturer.

### Real-time quantitative PCR

Total RNA from kidney and cultured cells was extracted by using TRIzol reagent (TransGene Biotech, China) and then transcribed into cDNA using the reverse transcription kit (TaKaRa, China), as instructed by the manufacturer. Quantitative real-time PCR was performed by using the TransStart Green qPCR SuperMix (TransGen Biotech). The GAPDH gene was used as a reference to normalize the data. The primer sequences used in the experiment are listed in Tables 1 and 2 in Supplementary materials.

### Immunoblot and immunoprecipitation

HK-2 cells and kidney tissues were lysed in cell lysis buffer (Beyotime Biotechnology) for Western blotting containing PMSF (Beyotime Biotechnology) on ice for at least 30 min. Cell lysis was centrifuged at 12,000 g for 10 min at 4 °C and the cell supernatants were collected. The following procedures were performed as previously described [18]. For immunoprecipitation, cell lysate was incubated with 1 µg of anti-TGF-β receptor I or II antibody and protein A/G agarose (Santa Cruz Biotechnology, USA) at 4 °C overnight, respectively. The eluted immunoprecipitants were resolved via SDS-PAGE to examine the terminal fucosylation level on TGF-β receptor I or II.

### Wound-healing assay

HK-2 cells (2 × 10^5^ cells per well) were seeded into 6-well plates (SORFA Life Science, China). Upon almost 100% confluence, the cell was scratched in a straight line by a sterile pipette tip and carefully washed to remove the detached cells. Subsequently, the cells were cultured with indicated treatment. Photographs of the scratch wound in different samples were recorded at 0 and 48 h.

### Cell viability assay

HK-2 cells (2 × 10^3^ cells per well) were seeded into 96-well plates. After overnight incubation, cells were treated with indicated stimulation for 24 or 48 h, and CCK-8 solution was added for 4 h [19]. Cell viability was determined by detection at an absorbance wavelength at 450 nm.

### Histological analysis

The kidney of each mouse was collected after sacrifice at day 14, fixed with 4% paraformaldehyde and embedded in paraffin. The tissues were cut into 5 μm thick sections and stained with hematoxylin and eosin (H&E), Masson’s Trichrome Stain to evaluate the severity of renal fibrosis. Foci of immune infiltrates were scored in the H&E sections according to the following criteria: 0 (no foci), 1 (< 2 foci per 200X field), 2 (2–4 foci per 200X field), or 3 (> 4 foci per 200X field) [20]. All histomorphological analysis was performed blindly.

### Immunofluorescence

Paraffin-embedded, formalin-fixed 5-µm-thick tissue sections or cells grown on coverslips in a 12-well plate were processed for staining with the primary antibodies. The following procedures to examine terminal fucosylation by UEA-I staining were performed as previously described [21, 22]. Images were obtained by laser scanning confocal microscopy (Olympus, FV1000, Japan).

### Statistical analysis

All values were expressed as mean ± SEM. Unpaired Student’s t-test was used for two-group comparisons, while one-way ANOVA followed by Tukey’s post-hoc tests was used for multiple group comparisons. P < 0.05 was considered significant. Statistics were calculated with GraphPad Prism version 8, GraphPad Software.

## Results

### FUT1-mediated terminal fucosylation is upregulated during the development of CKD

**Fig. 1:**
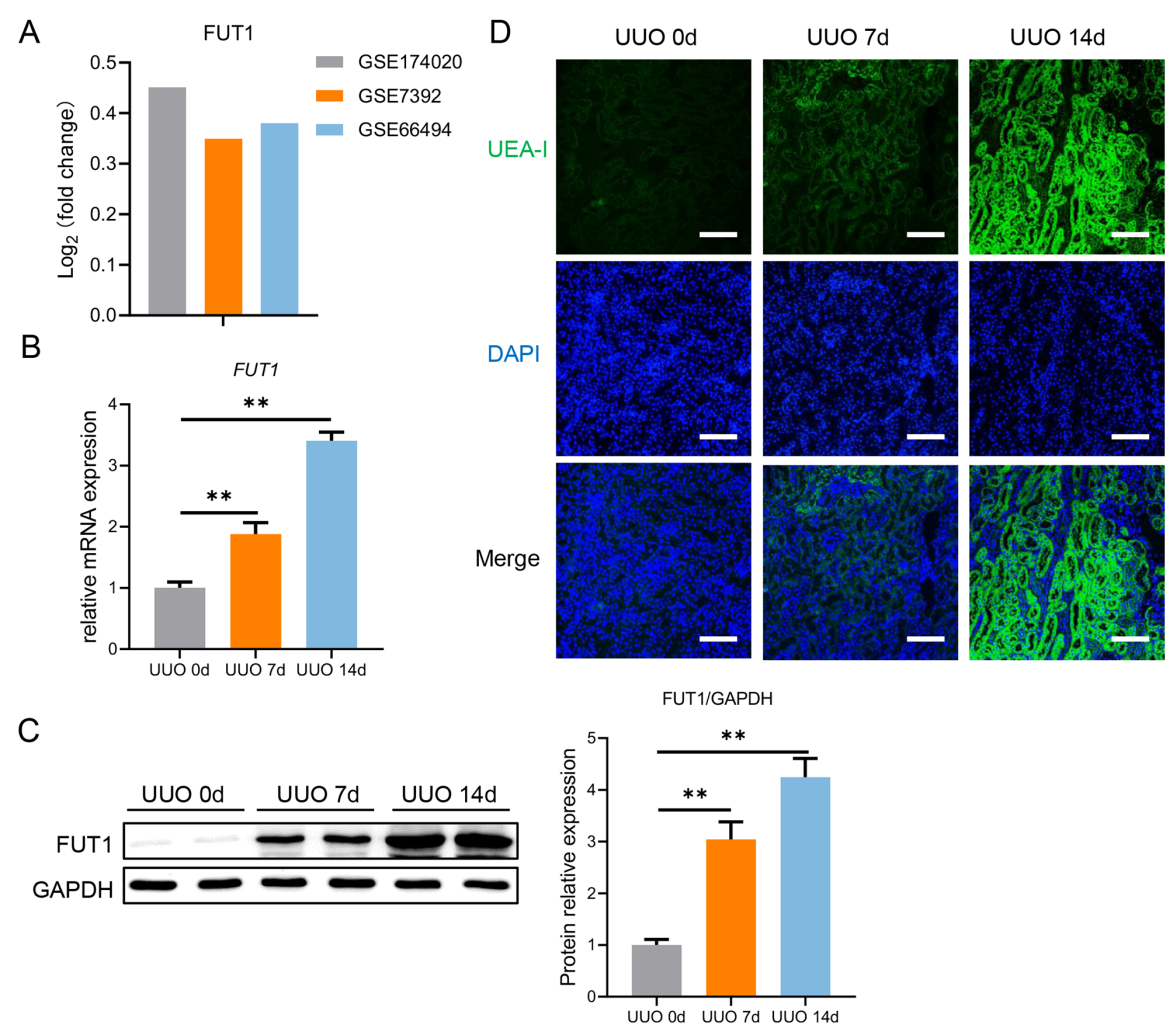
FUT1-mediated terminal fucosylation is upregulated during the development of CKD **(A)** Relative expression levels of FUT1 in CKD patients compared with healthy people in the GEO datasets. C57BL/6 mice (n = 5) established UUO model and sacrificed in day 0, 7 and 14. **(B)** The protein level of FUT1 in kidney tissues of mice was analyzed by western blotting. **(C)** The mRNA level of FUT1 in kidney tissues of mice was analyzed by qPCR. **(D)** The terminal fucosylation level (green) in kidney tissues of mice was analyzed by UEA-I staining. Scale bars = 200 μm. Values are expressed as the mean ± standard error; **P < 0.01

To explore the role of FUT1-mediated terminal fucosylation in chronic kidney diseases (CKD), especially in renal fibrosis, we first analyzed the expression of FUT1 based on public microarray datasets (GSE174020, GSE7392 and GSE66494) and found that FUT1 expression was higher in CKD patients compared to healthy people (Fig. 1A). Therefore, we applied a widely-accepted mice renal fibrosis model (UUO) [23] (Supplementary Fig. 1) to validate expression of FUT1. We observed that the level of FUT1 protein (Fig. 1B) as well as its mRNA expression (Fig. 1C) were remarkedly increased during kidney fibrogenesis, especially in day 14. Consistently, we observed that FUT1-mediated terminal fucosylation upregulated and mainly located in renal tubules via the detection of UEA-I (Fig. 1D). To sum up, these data together indicate that elevated FUT1-mediated terminal fucosylation is positively associated with the progression of renal fibrosis.

### FUT1 accelerates EMT to promote renal fibrosis in HK-2 cells

**Fig. 2:**
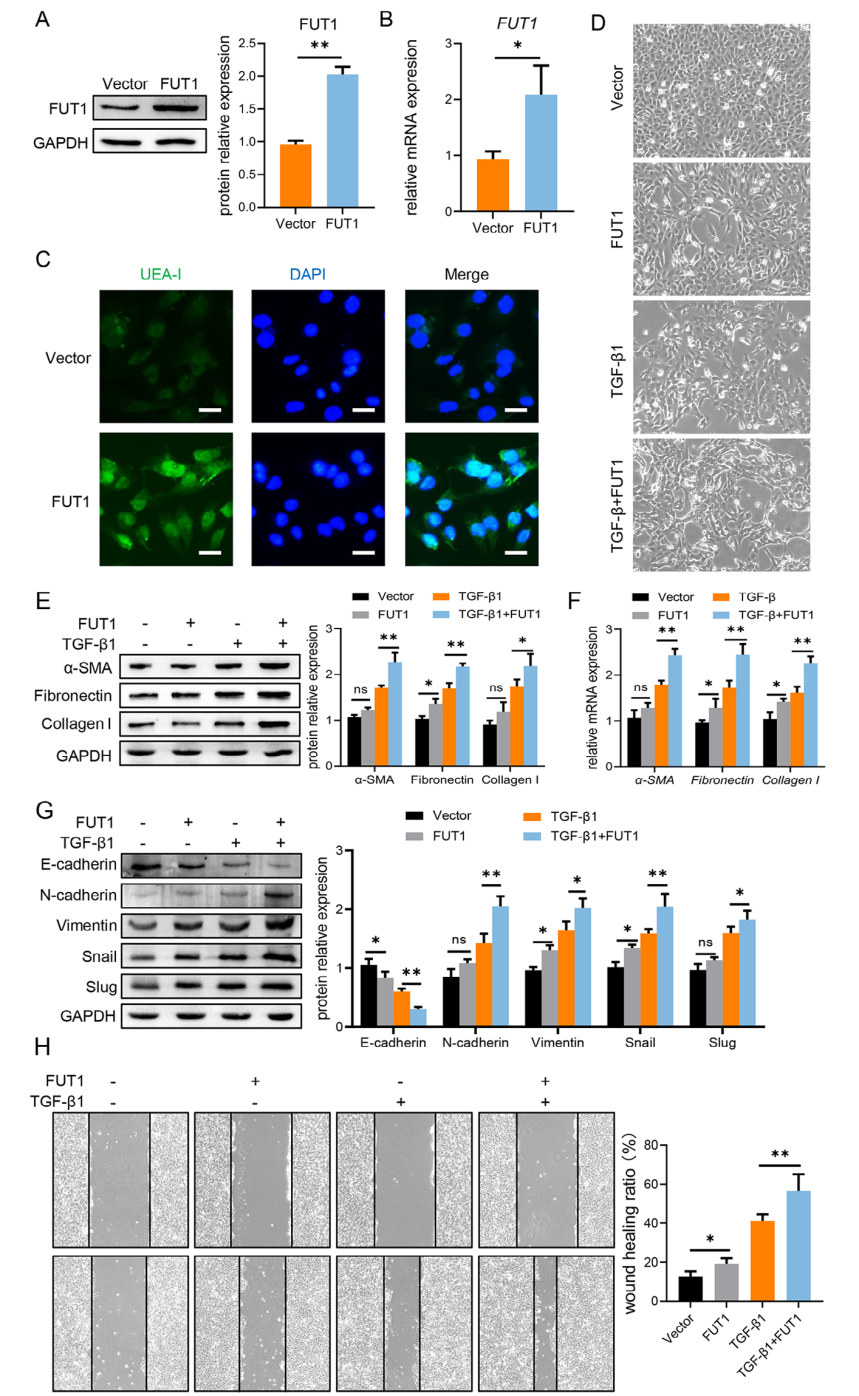
FUT1 accelerates EMT to promote renal fibrosis in HK-2 cells HK-2 cells were transfected FUT1 plasmid with or without TGF-β1 (10 ng/mL) for 48 h. **(A)** The protein level of FUT1 in HK-2. **(B)** The mRNA level of FUT1 in HK-2. **(C)** The terminal fucosylation level (green) in HK-2 cells was analyzed by UEA-I staining. **(D)** The morphology of HK-2 transfected FUT1 plasmid with or without TGF-β1. **(E)** The protein level of fibrosis-related molecules in HK-2 was analyzed by western blotting. **(F)** The mRNA level of fibrosis-related molecules in HK-2 was analyzed by qPCR. **(G)** The protein level of EMT-related molecules in HK-2 was analyzed by western blotting. **(H)** The ability of migration was examined in treated HK-2 by cell migration assay. Scale bars = 100 μm. Values are expressed as the mean ± standard error of the mean from three independent experiments; ns, not significant, *P < 0.05, **P < 0.01

The upregulation of FUT1 and terminal fucosylation expression in the renal tubules of mice kidney drove us to investigate its role in cell culture. To better understand the biological significance of FUT1-mediated terminal fucosylation during the pathogenesis of renal fibrosis, FUT1 over-expression was established in HK-2 cells (Fig. 2A, B). Accordingly, the over-expression of FUT1 raised terminal fucosylation in HK-2 (Fig. 2C). We found that the normal HK-2 cells exhibited typical cobblestone morphology with an epithelial phenotype while the stimulation of TGF-β1 drove HK-2 to develop a spindle shape and disassociate from neighboring cells. Strikingly, the over-expression of FUT1 could further promote TGF-β1-induced abnormal cellular morphology (Fig. 2D). What’s more, expression of fibrosis-related molecules (including α-SMA, fibronectin and collagen I) was elevated after transfected with FUT1 combined with TGF-β1 treatment (Fig. 2E, F). As EMT plays a pivotal role in the pathogenesis of renal fibrosis [6, 24, 25], we next assessed the expression of EMT-related molecules and found that FUT1 over-expression with TGF-β1 treatment could significantly increase EMT process in HK-2 cells (Fig. 2G). Accordingly, wound healing assay also suggested that the over-expression of FUT1 could promote cells migration via EMT (Fig. 2H). Notedly, overexpression FUT1 alone could slightly aggravate the development of fibrosis and EMT in HK-2 cells. In summary, these in vitro results reveal that FUT1-mediated terminal fucosylation could promote renal fibrosis via EMT process.

### Inhibition of terminal fucosylation ameliorates renal fibrosis severity via EMT in HK-2 cells

**Fig. 3:**
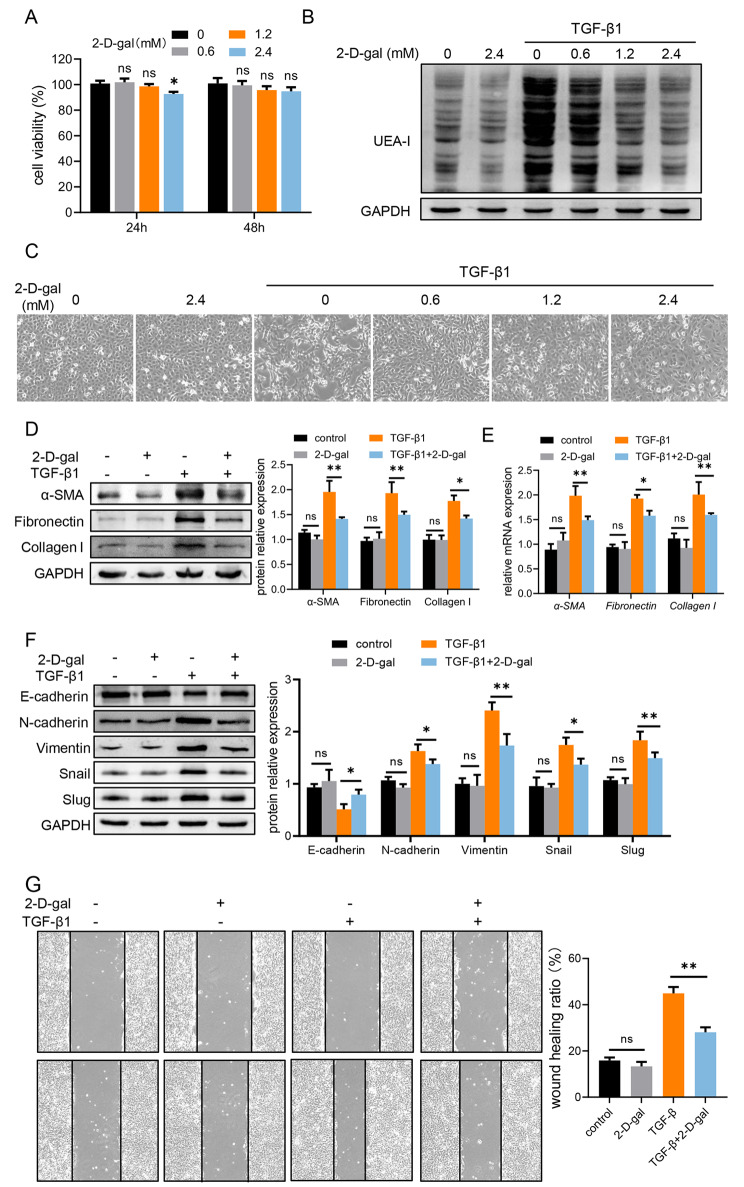
Inhibition of terminal fucosylation ameliorates renal fibrosis severity via EMT in HK-2 cells HK-2 were stimulated 2-D-gal (0.6, 1.2, 2.4 mM) and/or TGF-β1 for 24 or 48 h. **(A)** Cell viability was determined by CCK-8 assay. **(B)** The level of terminal fucosylation was evaluated in HK-2. **(C)** The morphology of HK-2. **(D)** The protein level of fibrosis-related molecules in HK-2 was analyzed by western blotting. **(E)** The mRNA level of fibrosis-related molecules in HK-2 was analyzed by qPCR. **(F)** The protein level of EMT-related molecules in HK-2 was analyzed by western blotting. **(G)** The ability of migration was examined in treated HK-2 by cell migration assay. Values are expressed as the mean ± standard error of the mean from three independent experiments; ns, not significant, *P < 0.05, **P < 0.01

As terminal fucosylation is positively associated with the kidney fibrosis, therefore, we applied 2-D-gal (a well-known potent α(1,2)-fucosylation inhibitor [26, 27]) to examine the effect of the terminal fucosylation inhibition in HK-2 cells. Firstly, we incubated HK-2 with different concentrations of 2-D-gal and CCK-8 assay suggested 2-D-gal hardly affected the viability of HK-2 cells (except 2.4 mM 2-D-gal for 24 h) (Fig. 3A). Furthermore, we observed that 2-D-gal suppressed terminal fucosylation in a dose-dependent manner but with an effective plateau or saturation at concentrations higher than 1.2 mM (Fig. 3B), so we chose 1.2 mM 2-D-gal for the following experiments. It is worthy to mention that TGF-β1 is able to increase FUT1 expression (Supplementary Fig. 2A, B) and terminal fucosylation in HK-2 (Fig. 3B) in a dose-dependent manner. After the treatment of 2-D-gal, HK-2 exhibited cobblestone-like cell morphology resembling cell under the normal condition rather than under TGF-β1 stimulation (Fig. 3C). Accordingly, expression of fibrosis-related molecules was all decreased after treating with 2-D-gal (Fig. 3D, E). Previous results in Fig. 2E, G indicated FUT1-mediated terminal fucosylation plays a key role in EMT during renal fibrosis, we then investigated whether terminal fucosylation inhibitor downregulates EMT to alleviate renal fibrosis. 2-D-gal indeed inhibited EMT process in HK-2, confirmed by the examination of EMT-related protein expression as well as healing wound ratio (Fig. 3F, G). Together, these data indicate inhibition of terminal fucosylation by 2-D-gal suppresses renal fibrogenesis via targeting EMT process.

### Suppressing terminal fucosylation on TGF-β receptor I and II attenuates fibrosis in HK-2 cells

**Fig. 4:**
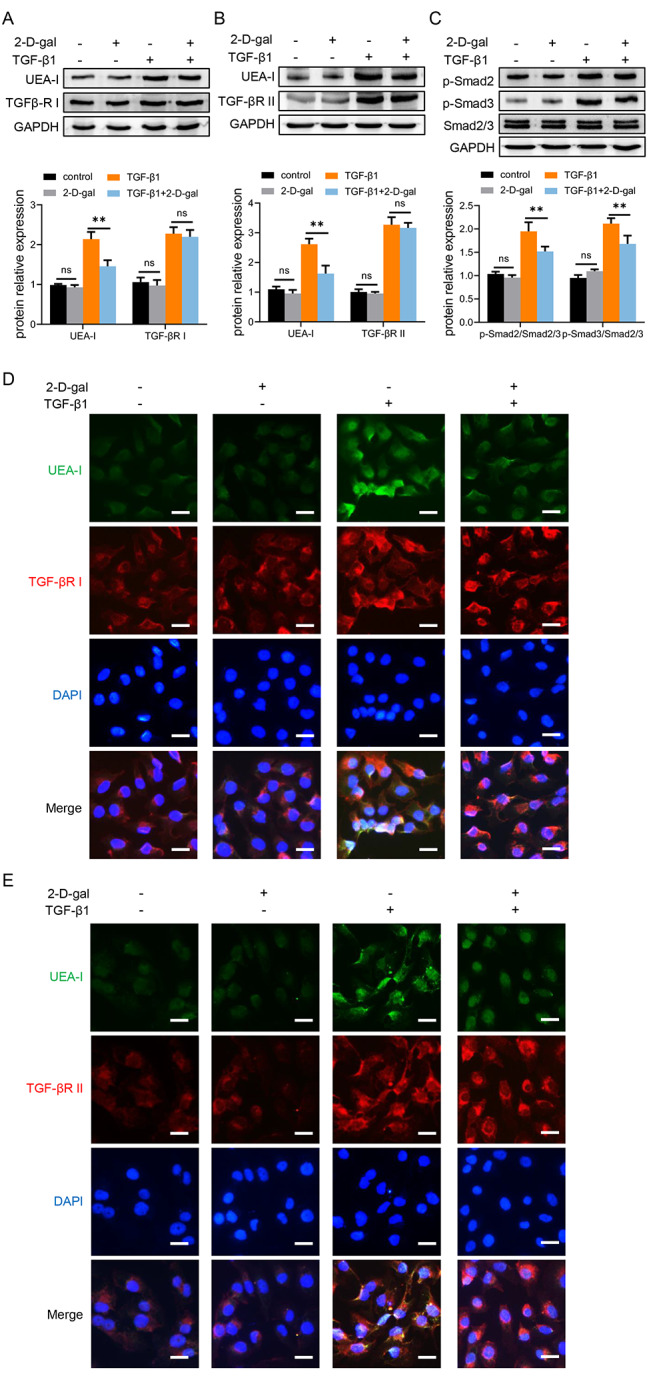
Suppressing terminal fucosylation on TGF-β receptor I and II attenuates fibrosis in HK-2 cells HK-2 were stimulated 2-D-gal (1.2 mM) and/or TGF-β1 for 48 h. **(A)** The terminal fucosylation level on TGF-βR I was analyzed by Co-IP. **(B)** The terminal fucosylation level on TGF-βR II was analyzed by Co-IP. **(C)** The protein level of p-Smad2, p-Smad3 and Smad2/3 in HK-2 was analyzed by western blotting. (**D**) Representative photomicrographs of UEA-I (green) and TGF-βR I (red) expression by immunofluorescence analysis. (**E**) Representative photomicrographs of UEA-I (green) and TGF-βR II (red) expression by immunofluorescence analysis. Scale bars = 100 μm. Values are expressed as the mean ± standard error of the mean from three independent experiments; ns, not significant, **P < 0.01

Emerging studies have demonstrated that TGF-β receptor is closely involved with fibrosis progression via EMT process [28]. Moreover, the aberrant fucosylation on TGF-β receptor is suggested to activate its biological function [29, 30]. Therefore, we examined the terminal fucosylation level on TGF-β receptor I and II respectively via Co-IP assay. We found that TGF-β1 stimulation could significantly upregulate the expression of TGF-β receptors as well as the terminal fucosylation on both TGF-β receptor I and II. However, terminal fucosylation inhibitor significantly inhibited the terminal fucosylation of TGF-βR I and II induced by TGF-β1 stimulation (Fig. 4A, B). It is well-known that Smad signaling is a classic downstream pathway in response to TGF-β1, so we investigated the expression of Smad signaling and found that 2-D-gal remarkably suppressed the phosphorylation of Smad2 and Smad3 (Fig. 4C). Accordingly, immunofluorescence also indicated that the terminal fucosylation level on both TGF-βR I and II is downregulated by 2-D-gal (Fig. 4D, E). In summary, terminal fucosylation on TGF-β receptor I and II increases during fibrogenesis and is significantly suppressed by 2-D-gal, resulting in inhibition of TGF-β/Smad pathway and subsequent EMT.

### 2-D-gal inhibits EMT via TGF-β/Smad pathway to ameliorate renal fibrosis

**Fig. 5:**
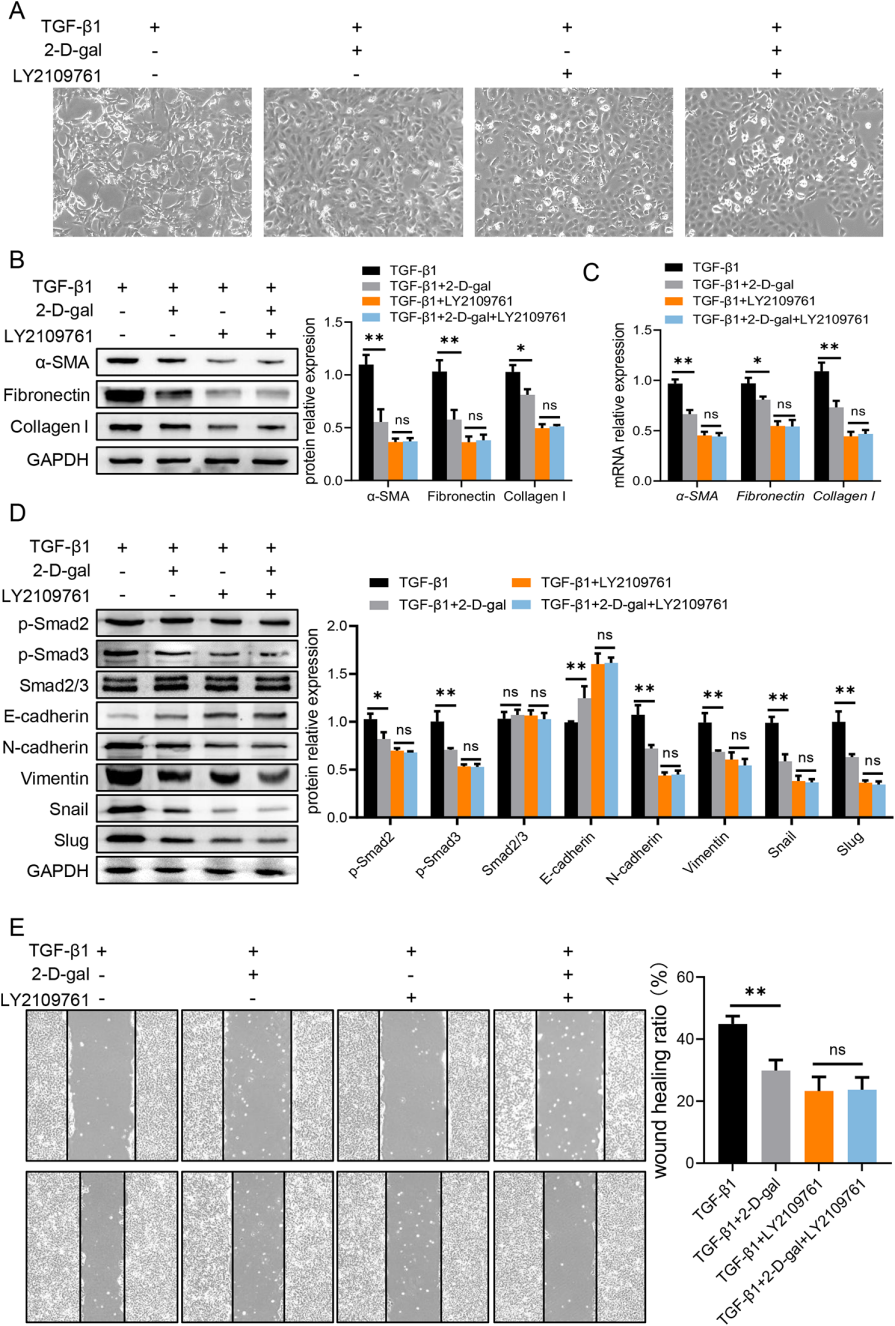
2-D-gal inhibits EMT via TGF-β/Smad pathway to ameliorate renal fibrosis HK-2 cells were pre-treated with LY2109761 for 2 h ahead of treatment with 2-D-gal and TGF-β1. (**A**) The morphology of HK-2. (**B**) The protein level of fibrosis-related molecules in HK-2 was analyzed by western blotting. (**C**) The mRNA level of fibrosis-related molecules in HK-2 was analyzed by qPCR. (**D**) The protein level of EMT-related molecules in HK-2 was analyzed by western blotting. (**E**) The ability of migration was examined in treated HK-2 by cell migration assay. Values are expressed as the mean ± standard error of the mean from three independent experiments; ns, not significant, *P < 0.05, **P < 0.01

Next, we applied TGF-β signaling specific inhibitor, LY2109761 [31, 32], to further validate the targeting pathway of 2-D-gal during renal fibrogenesis. Here, we observed that the pretreatment of LY2109761 induced cobblestone-like cell morphology and the differences between 2-D-gal treated or untreated cells were abolished (Fig. 5A). Furthermore, LY2109761 incubation could also eliminate the differences of fibrosis-related expression between 2-D-gal treated or untreated HK-2 cells (Fig. 5B, C). The EMT-related molecules as well as wound healing ratio was significantly inhibited by LY2109761 and became comparable between 2-D-gal treated or untreated cells (Fig. 5D, E). Taken together, 2-D-gal exerts anti-fibrotic function via the suppression of TGF-β/Smad pathway.

### 2-D-gal ameliorates renal fibrosis in UUO-induced renal fibrosis mice

**Fig. 6:**
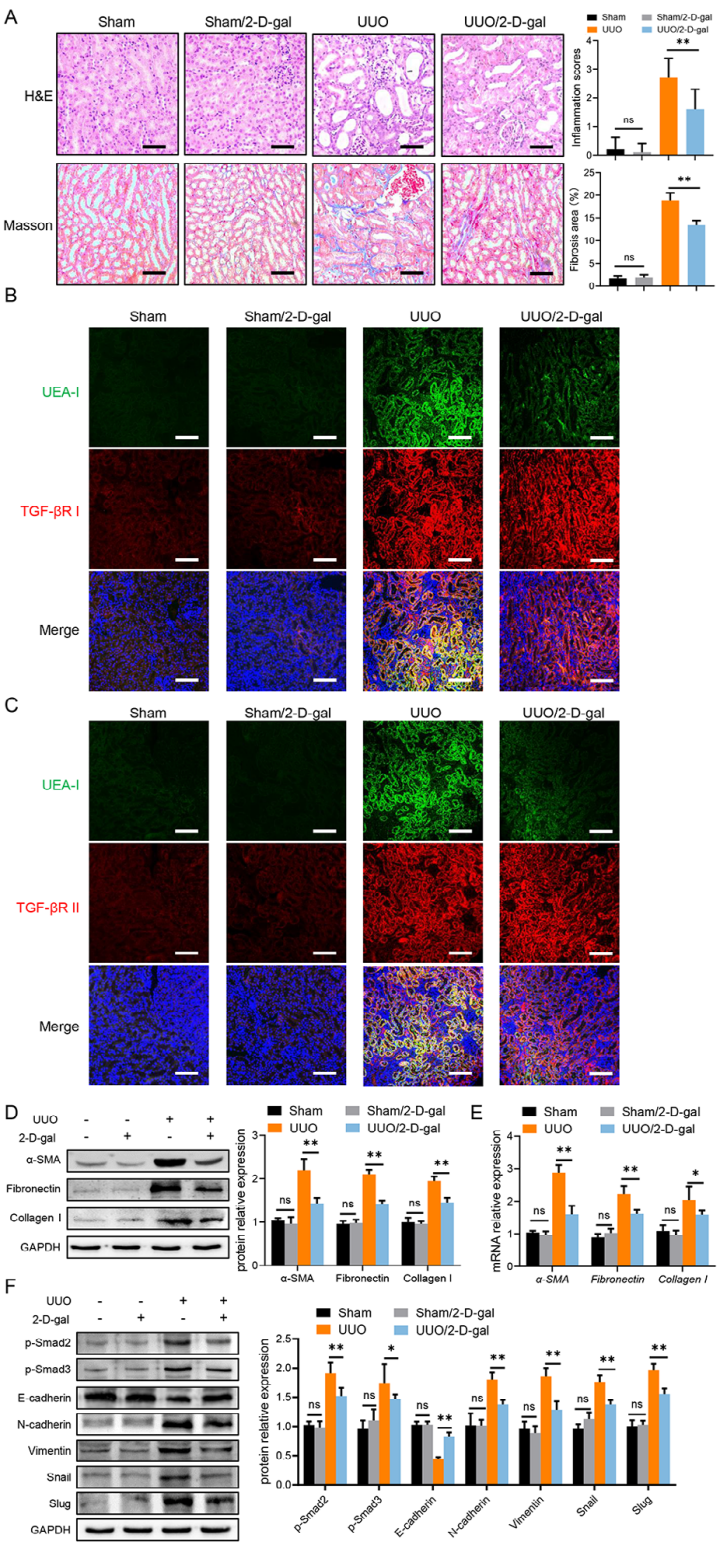
2-D-gal ameliorates renal fibrosis in UUO-induced renal fibrosis mice C57BL/6 mice (n = 5) received UUO to establish renal fibrosis with or without 2-D-gal application via intraperitoneal injection (250 mg/kg) every other day. (**A**) Representative photomicrographs of H&E and Masson’s Trichrome Stain in kidney tissues of mice. (**B**) Representative photomicrographs of UEA-I (green) and TGF-βR I (red) expression in mice kidney by immunofluorescence analysis. (**C**) Representative photomicrographs of UEA-I (green) and TGF-βR II (red) expression in mice kidney by immunofluorescence analysis. (**D**) The protein level of fibrosis-related molecules in kidney tissues of mice was analyzed by western blotting. (**E**) The mRNA level of fibrosis-related molecules in HK-2 was analyzed by qPCR. (**F**) The protein level of p-Smad2, p-Smad3 and EMT-related molecules in kidney tissues of mice was analyzed by western blotting. Scale bars = 200 μm. Values are expressed as the mean ± standard error; ns, not significant, *P < 0.05, **P < 0.01

According to the above in vitro experimental results, we are intrigued to examine the effect of 2-D-gal treatment against renal fibrosis in vivo. Here, based on previous studies [13, 33], we applied 2-D-gal via intraperitoneal injection (250 mg/kg) every other day during UUO-induced renal fibrosis mice until day 14. Notably, 2-D-gal could reduce the inflammatory infiltration cells, ECM deposition and improve kidney destruction in UUO-induced mice renal fibrosis (Fig. 6A). What’s more, the co-staining of UEA-I and TGF-βR I or II showed that 2-D-gal significantly inhibited the terminal fucosylation on TGF-β receptors, supported by results in HK-2 cells (Fig. 6B, C). Moreover, the expression of fibrosis-related and EMT-related molecules were regulated by 2-D-gal treatment, being similar with in vitro results (Fig. 6D-F). To sum up, 2-D-gal in vivo application markedly ameliorates renal fibrosis, indicating terminal fucosylation inhibitor is a promising clinical treatment in the future.

## Discussion

Renal fibrosis is a universal pathological development across chronic kidney diseases in terminal stage [34]. Recently, the aberrant level of terminal fucosylation is found to be closely related with the development of multiple diseases, including liver cancer [33], rheumatoid arthritis [35] and so on. Therefore, we were intrigued to investigate the correlation between FUT1-mediated terminal fucosylation and kidney fibrosis as well as the potential application of potent selective α (1, 2)-fucosylation inhibitor, 2-D-gal, for preventing the deterioration of kidney fibrosis. In the current study, we first discovered that aberrant terminal fucosylation in patients with CKD as well as during the development of renal fibrosis in mice. What’s more, our results also demonstrated the protective effect of 2-D-gal via suppressing TGFβ/Smad pathway in UUO-induced renal fibrosis mice and TGF-β1-treated human kidney proximal tubular epithelial cells. Overall, our findings did emphasize the significant role of terminal fucosylation in the pathogenesis of renal fibrosis and find a possible drug to against renal fibrosis.

Emerging evidence revealed that aberrant fucosylation level contributes to the progression of fibrogenesis [11, 36, 37]. In our current investigation, we are the first to found that patients with CKD expressed higher terminal fucosylation compared with healthy people, which is supported by the FUT1 expression and UEA-I level in UUO-induced renal fibrogenesis mice. Furthermore, single FUT1 over-expression mildly elevated fibrosis-related molecules expression, while in combination with TGF-β1 could notedly promote renal fibrosis in HK-2 cells. Interestingly, in vitro experiments also suggested that TGF-β1 dose-dependently induced terminal fucosylation via increased expression of FUT1, which is consistent with previous study showing FUT1 expression in response to TGF-β1 in ovarian cancer [38]. However, more experiments have to be conducted to further verify the underlying regulatory mechanism between TGF-β1 and FUT1 in the future. Together, these present results suggest that FUT1-mediated terminal fucosylation deteriorates renal fibrosis both in vitro and in vivo.

EMT is an indispensable mechanism contributing to the progression of renal fibrogenesis, where fibroblasts transformed from epithelial cells produce ECM to reconstruct kidney microenvironment [39–41]. It is worthwhile to mention that the role of EMT during the process of renal fibrosis has been questioned, because the types of matrix-producing cells and the exact role of tubule epithelium remain partly unclear [42]. Here, we observed that FUT1 over-expression combined with TGF-β1 treatment in HK-2 cells significantly drove EMT process to promote fibrosis progress. Therefore, we applied 2-D-gal to investigate whether terminal fucosylation inhibitor is a potential candidate to curb fibrosis development via EMT. We found that treatment of 2-D-gal indeed decreased fibrotic genes expression and maintained HK-2 epithelial-like morphology, accompanied with regulation of EMT-related protein expression (including upregulation of E-cadherin, while downregulation of N-cadherin, Vimentin, Slug and Snail) as well as suppression of wound healing ability. Accordingly, in vivo experiments also confirmed that 2-D-gal ameliorated mice renal fibrosis via modulating the course of EMT. In summary, our current findings provide preliminary evidence that proper application of terminal fucosylation inhibitor, 2-D-gal, improves the renal fibrosis condition via inhibiting EMT process.

As TGF-β1 is a key regulator of EMT process and ECM deposition, TGF-β receptors are indispensable in the development of renal fibrosis [43] and inhibition of either TGF-β receptor I or II remarkably hinders EMT process [44, 45]. Recent studies have demonstrated that TGF-β receptors as glycoproteins are modified by core fucosylation which is dependent on FUT8 [11, 29]. Here, aberrant core fucosylation was found in UUO-induced renal fibrosis mice and TGF-β1-induced HK-2 cells, however, suppression of core fucosylation on TGF-β receptor I and II significantly attenuated the progression of renal fibrosis. Accordingly, the terminal fucosylation on TGF-β receptor I and II are upregulated under the treatment of TGF-β1, while terminal fucosylation inhibitor significantly inhibited the terminal fucosylation on both TGF-β receptor I and II, further supported by the results of 2-D-gal treatment in vivo. After TGF-β1 activation, TGF-β receptor I and II formed heteromeric complex which leads to increase of phosphorylated Smad2 and Smad3, subsequently promoting EMT via transcriptional regulation [8]. As results obtained in the present study show, phosphorylation of Smad2 and Smad3 in TGF-β stimulated HK-2 cells was increased, while phosphorylation of Smad2 and Smad3 was effectively suppressed by 2-D-gal treatment. In consistent with this, the anti-fibrotic and anti-EMT effect of 2-D-gal was abolished upon pretreatment with TGF-β receptor inhibitor confirming that TGF-β/Smad signaling is responsible for the inhibitory effect of 2-D-gal on HK-2 cells. Correspondingly, in vivo results also indicate that 2-D-gal alleviates renal fibrosis via TGF-β/Smad pathway.

## Conclusion

In summary, our study first finds out that aberrant terminal fucosylation contributes to renal fibrosis and CKD. Thus, inhibition of terminal fucosylation by 2-D-gal improves the renal fibrosis condition, mainly targeting TGF-β1/Smad signaling pathway and subsequent EMT process in vivo and in vitro. Collectively, our present work unravels the clinical significance of terminal fucosylation during renal fibrosis progression and demonstrates the application of terminal fucosylation inhibitor, such as 2-D-gal, is a novel potential therapeutic strategy for treating renal fibrogenesis.

## Electronic supplementary material

Below is the link to the electronic supplementary material.

Supplementary Material 1

## Data Availability

Data will be made available on reasonable request.

## Declarations

### Abbreviations

2-D-gal, 2-Deoxy-D-galactose; CKD, chronic kidney disease; DMEM, Dulbecco’s modified Eagle’s medium; ECM, extracellular matrix; EMT, epithelial mesenchymal transition; FBS, fetal bovine serum; FUT, fucosyltransferases; H&E, hematoxylin and eosin; TGF-β, transforming growth factor-β; UEA-I, ulex europaeus agglutinin-I; UUO, unilateral ureteral obstruction.

**Ethics approval and consent to participate**.

All animal experiments were approved by the Welfare and Ethical Committee for Experimental Animal Care of Southern Medical University.

**Consent for publication**.

All the authors give consent.
