# Supplementary material for: FUT1-mediated terminal fucosylation acts as a new target to attenuate renal fibrosis
Source: Mol Med. 2023 Apr 21;29:55. doi: 10.1186/s10020-023-00639-0 (PMC10122342; doi:10.1186/s10020-023-00639-0)
Supplement: Supplementary file 1 — Supplementary Material 1 [file 10020_2023_639_MOESM1_ESM.docx]

Supplementary table 1. The RT-PCR primers used in this study (Mouse)

|  | Forward primer (5’-3’) | Reverse primer (5’-3’) |
| --- | --- | --- |
| FUT1 | CAGCTCTGCCTGACATTTCTG | AGCAGGTGATAGTCTGAACACA |
| α-SMA | GTCCCAGACATCAGGGAGTAA | TCGGATACTTCAGCGTCAGGA |
| Fibronectin | ATGTGGACCCCTCCTGATAGT | GCCCAGTGATTTCAGCAAAGG |
| Collagen I | GCTCCTCTTAGGGGCCACT | CCACGTCTCACCATTGGGG |
| GAPDH | AGGTCGGTGTGAACGGATTTG | TGTAGACCATGTAGTTGAGGTCA |

Supplementary table 2. The RT-PCR primers used in this study (Human)

|  | Forward primer (5’-3’) | Reverse primer (5’-3’) |
| --- | --- | --- |
| FUT1 | CTTCCTGCTAGTCTGTGTCCT | ATTGGGGTAGACAGTCCAGGT |
| α-SMA | AAAAGACAGCTACGTGGGTGA | GCCATGTTCTATCGGGTACTTC |
| Fibronectin | AGGAAGCCGAGGTTTTAACTG | AGGACGCTCATAAGTGTCACC |
| Collagen I | ATCAACCGGAGGAATTTCCGT | CACCAGGACGACCAGGTTTTC |
| GAPDH | CTGGGCTACACTGAGCACC | AAGTGGTCGTTGAGGGCAATG |

**Supplementary Figure1**


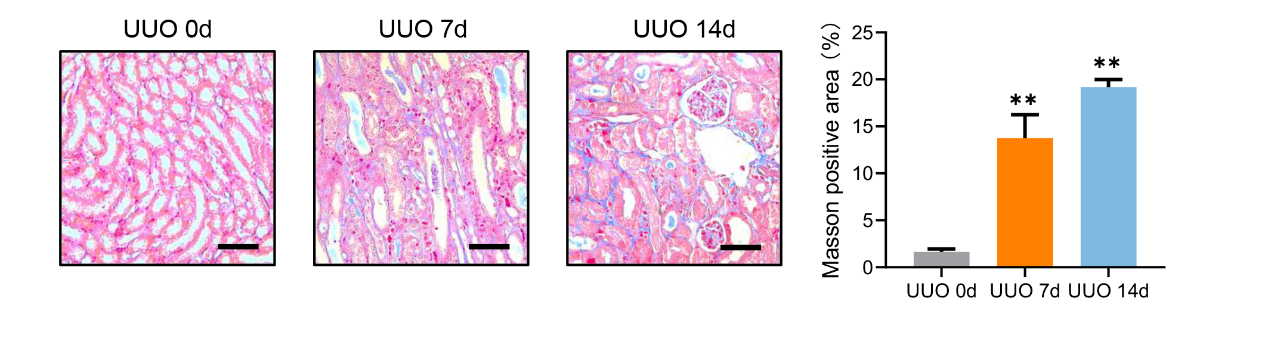


**The establishment of UUO mice model.** Mice were received UUO and sacrificed on day 0, 7 and 14. Representative photomicrographs of Masson’s Trichrome Stain in kidney tissues of mice. Scale bars = 200 μm. Values are expressed as the mean ± standard error; *P < 0.05, **P < 0.01.

**Supplementary Figure2**

**
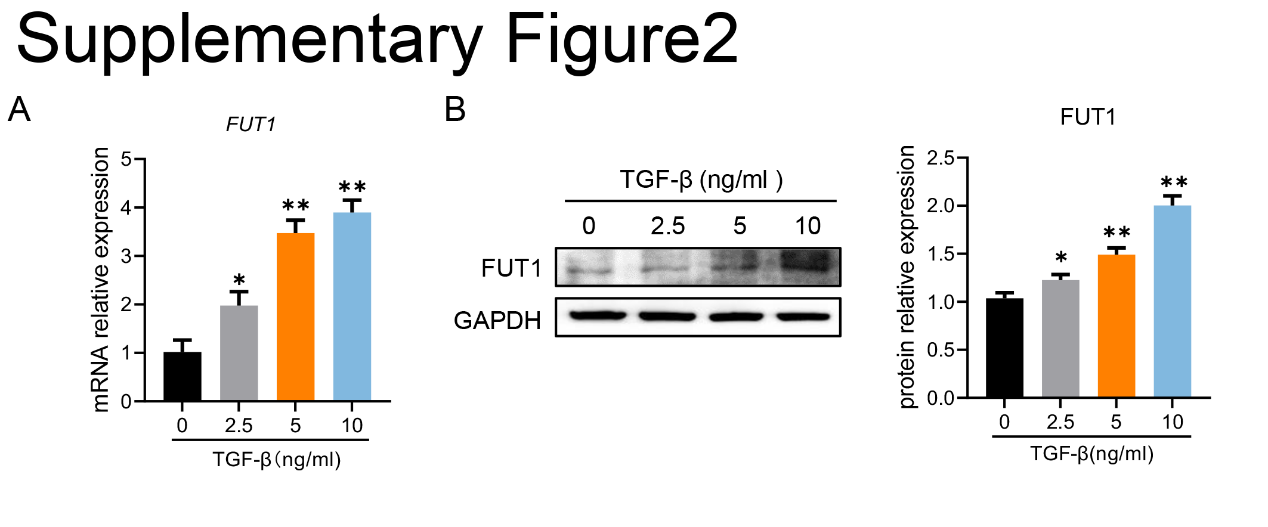
**

**TGF-β1 induces FUT1 expression.** HK-2 cells were stimulated with different concentrations of TGF-β1 for 48 hours. (A) The mRNA expression of FUT1 was analyzed by qPCR. (B) The protein expression of FUT was analyzed by western blotting. Values are expressed as the mean ± standard error of the mean from three independent experiments; *P < 0.05, **P < 0.01.
